# Editorial: Detection Nanodevices for Infectious Diseases

**DOI:** 10.3389/fbioe.2022.962746

**Published:** 2022-06-29

**Authors:** Yung-Chih Wang, Yi-Tzu Lee, Koji Matsuura, Xinyu Liu, Chao-Min Cheng

**Affiliations:** ^1^ Division of Infectious Diseases and Tropical Medicine, Department of Internal Medicine, Tri-Service General Hospital, National Defense Medical Center, Taipei, Taiwan; ^2^ Department of Emergency Medicine, Taipei Veterans General Hospital, Taipei, Taiwan; ^3^ Faculty of Medicine, School of Medicine, National Yang-Ming University, Taipei, Taiwan; ^4^ Department of Biomedical Engineering, Faculty of Engineering, Okayama University of Science, Okayama, Japan; ^5^ Department of Mechanical and Industrial Engineering, University of Toronto, Toronto, ON, Canada; ^6^ Institute of Biomedical Engineering, National Tsing Hua University, Hsinchu, Taiwan

**Keywords:** aptamer, infectious diseases, nanocarriers, paper-based test strip, point-of-care testing, spectrum-based optical reader

Infectious diseases are transmissible diseases caused by pathogenic microorganisms, such as bacteria, viruses, parasites or fungi, which confer to morbidities and mortalities in humans all over the world. The challenge of the managements of infectious diseases involves both diagnosis and treatment counterparts. Early recognition of the presence of causative microorganism is the key factor to implement an optimal treatment of the infectious diseases. The application of bioengineering and biotechnology makes the rapid and precise detection of infectious agents possible. During the pandemic, such as COVID-19 infections, these tools will be necessary for telemedicine (Wang et al., 2020). In addition, a more rapid diagnosis for drug-resistant pathogens can help optimal therapy for these infectious diseases. These will further improve the clinical treatment and patient outcomes.

Apart from the early diagnosis, the timely treatment is important for the treatment of infectious diseases. However, the emergence of drug-resistance pathogen has limited the effects of current antibiotics. The WHO has listed several priority pathogens for which new antimicrobial is urgently needed (Tacconelli et al., 2018). As the development of new antimicrobials is time-consuming or expensive, the discovery of innovative approaches for the treatment of infectious diseases catches much attention. There are several modalities found to be used for the management of infectious diseases, such as bacteriophages, microbiome-modulating agents, antibacterial antibodies, and nanomaterials (Shemyakin et al., 2020; Iskandar et al., 2022).

The goal of this Research Topic is to introduce promising and novel research trends in the detection devices for infectious diseases field. In addition, the innovative modalities for the therapeutic agents against infectious diseases are provided. Overall, we have collected five research articles focused on the diagnosis or treatment of infectious diseases, including two original articles, two reviews, and one research report, contributed by the experts in this field.

Lin et al., in their work, designed an interleukin-6 (IL-6) paper-based test strip that used colloidal gold-conjugated antibodies to detect human IL-6 protein. While IL-6 has been found to be associated with cytokine storm following infection with avian influenza A H5N1 (De Jong et al., 2006), it has been demonstrated to be correlated with sepsis severity and mortality (Ma et al., 2016). Their paper-based test strips can detect IL-6 *via* lateral flow immunoassay. They successfully enhanced the detection with the aid of a spectrum-based optical reader. In addition, they validated that their system could be applied for differentiating between severe and mild influenza using serum obtained from children. Their point-of-care (POC) system harbored easy-to-use, rapid, high detection performance, and low-cost properties. It potentially could promote early detection of disease severity and target antiviral therapy for children with influenza virus infection.

Following the same methodology, Wang et al. detected the IL-6 level from serum of those with COVID-19. IL-6 have been found to be associated with respiratory failure in those with COVID-19 (Leisman et al., 2020). As Omicron variant causes less severe disease than infection with prior variants, it is important to identify those at risk for respiratory failure during the COVID-19 pandemic era. The IL-6 test strip exhibited excellent sensitivity and specificity for the recognition of patients with mechanical ventilation using a cutoff value of 76.85 pg/ml. The incorporation of an IL-6 lateral flow immunoassay test strip and a spectrum-based optical reader makes the POC diagnostic device a promising tool for early recognition of patients at risk of respiratory failure requiring mechanical ventilation. This would assist clinician for timely diagnosis and reduce the delay management of the disease.

Gram-negative bacteria can shed outer-membrane vesicles (OMVs) containing membrane-bound proteins, toxins, enzymes, DNA, RNA, and peptidoglycan (Kaparakis-Liaskos and Ferrero, 2015), which play important roles in the pathogenesis in infectious diseases (Schwechheimer and Kuehn, 2015). Apart from bacteria, human cells can release nanovesicles, named exosomes, which can be used as biomarkers for disease diagnosis. Lai et al. also integrated a paper-based immunoaffinity and a paper-based silica device to develop a novel system. This designed paper-based system has successfully employed to capture exosomes and exosomal nucleic acids from various biological samples. This approach can provide a novel method for capturing exosomes and nucleic acids using easily operated and cheap equipment. It has the potential to replace traditional methodologies for POC testing and could provide clinical applications, especially in low-resources areas.

Aptamers are a class of single-stranded DNA or RNA molecules that are selected for binding to a specific target (Zhou and Rossi, 2017; Yoon and Rossi, 2018), which are synthetic screened *in vitro* by a selection procedure known as systematic evolution of ligands by exponential enrichment (SELEX) (Tuerk and Gold, 1990). With the advance of biotechnology, several novel SELEX approaches for aptamer selection have been developed. Xu et al. provided a comprehensive review of aptamer-based techniques and SELEX technologies for the diagnosis and treatment of infectious diseases. They summarized the pros and cons of the SELEX techniques and listed the current developed aptamers in the detections of various pathogens. They also proposed the future perspectives for the application of aptamer in precision medicine for the treatment of infectious diseases. These state-of-the-art technologies could provide a quantum leap for the diagnosis and treatment of the drug-resistance as well as the emerging pathogens.

Yang et al., in their review paper, comprehensively summarized the recent developments for future nano-antibiotics. They demonstrated the mechanisms of antibiotic resistance developed in bacteria including intrinsic and acquired resistance. This gives us a better understanding on the actions of resistance and help us to tackle the problem. Although several novel approaches have been explored to combat the antibiotic resistance, such as antimicrobial peptides (Lazzaro et al., 2020), bacteriophages (Torres-Barceló, 2018), phytochemicals (Ayaz et al., 2019), and nanomaterials (Lee et al., 2019), special attention has been paid in highlighting the applications of surface engineered nano-cargos for antibiotic resistance nanomedicine. They summarized different nanoparticle-based antibiotic delivery strategies for skin and subcutaneous infections tested in animal studies. The strength and limitations of nanocarriers for antibiotics were evaluated. This review provided a thorough vision of nano-cargos for the potential applications on multidrug-resistant bacterial infections.

In summary, the articles collected in this Research Topic demonstrate the advances of techniques for the development of detection and therapeutic modalities for infectious diseases. The lateral flow immunoassay test strip coupled spectrum-based optical readers, aptamer-based technologies, and the nanocarriers introduced in these articles provide valuable information. Although there are still many challenges facing the infectious diseases, we believe that the information included in this Research Topic may shed light on the battle against infectious diseases.
